# Isolation and Characterization of *APETALA3* Orthologs and Promoters from the Distylous *Fagopyrum esculentum*

**DOI:** 10.3390/plants10081644

**Published:** 2021-08-10

**Authors:** Lingtian Zeng, Jiao Zhang, Xuan Wang, Zhixiong Liu

**Affiliations:** College of Horticulture and Gardening, Yangtze University, Jingzhou 434025, China; 202072800@yangtzeu.edu.cn (L.Z.); 201871411@yangtzeu.edu.cn (J.Z.); 201872469@yangtzeu.edu.cn (X.W.)

**Keywords:** *APETALA3*-like gene, *Fagopyrum esculentum*, MADS-box gene, promoter, stamen development

## Abstract

Common buckwheat (*Fagopyrum esculentum*) produces distylous flowers with undifferentiated petaloid tepals, which makes it obviously different from flowers of model species. In model species *Arabidopsis*, *APETALA3* (*AP3*) is expressed in petal and stamen and specifies petal and stamen identities during flower development. Combining with our previous studies, we found that small-scale gene duplication (GD) event and alternative splicing (AS) of common buckwheat *AP3* orthologs resulted in *FaesAP3_1*, *FaesAP3_2* and *FaesAP3_2a*. *FaesAP3_2* and *FaesAP3_2a* were mainly expressed in the stamen of thrum and pin flower. Promoters functional analysis suggested that intense GUS staining was observed in the whole stamen in *pFaesAP3_2::GUS* transgenic *Arabidopsis*, while intense GUS staining was observed only in the filament of stamen in *pFaesAP3_1::GUS* transgenic *Arabidopsis.* These suggested that *FaesAP3_1* and *FaesAP3_2* had overlapping functions in specifying stamen filament identity and work together to determine normal stamen development. Additionally, *FaesAP3_2* and *FaesAP3_2a* owned the similar ability to rescue stamen development of *Arabidopsis ap3-3* mutant, although AS resulted in a frameshift mutation and consequent omission of the complete PI-derived motif and euAP3 motif of *FaesAP3_2a*. These suggested that the MIK region of *AP3*-like proteins was crucial for determining stamen identity, while the function of *AP3*-like proteins in specifying petal identity was gradually obtained after *AP3* Orthologs acquiring a novel C-terminal euAP3 motif during the evolution of core eudicots. Our results also provide a clue to understanding the early evolution of the functional specificity of euAP3-type proteins involving in floral organ development in core eudicots, and also suggested that *FaesAP3_2* holds the potential application for biotechnical engineering to develop a sterile male line of *F. esculentum.*

## 1. Introduction

**Figure 1:**
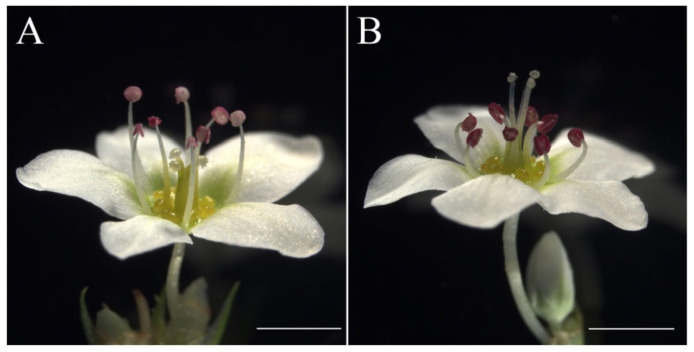
Distylous flower of *F. esculentum.* (**A**) Thrum flower with short pistil and long stamen; (**B**) pin flower with long pistil and short stamen. Scale bar = 2 mm.

Common buckwheat (*Fagopyrum esculentum*) is a gluten-free pseudo-cereal crop mainly cultivated in Europe, North America and Asian for human consumption and forage [1,2]. Buckwheat grains are rich in bioactive compounds, such as rutin, quercetin, polysaccharides and dietary fiber, etc., but with low-calories. Hence, common buckwheat is increasingly demanded and recognized for its highly effective as a functional food with health benefits and illness prevention throughout recent years [1]. However, common buckwheat is an obligate outcrossing crop with heteromorphic self-incompatibility due to its distylous flowers, with population being equally composed of plants with thrum flowers (short pistil combined with long stamen and large pollen grains) and plants with pin flowers (long pistil combined with short stamen and small pollen grains) (Figure 1), and legitimate cross-pollinations occur strictly between anthers and stigmas of equivalent height in different floral morphs, which make it hard for hybrid breeding and result in low yield [3,4]. Improving the yield stability and efficiency of breeding programs requires a better understanding the developmental genetics of distylous flowers in common buckwheat. Exploring the underlying molecular mechanism of heteromorphic self-incompatibility of common buckwheat has continued for a quarter of a century and has made considerable progress in understanding of the molecular basis of heteromorphic SI in recent years [4,5,6]. However, the molecular mechanisms responsible for the form and development of the dimorph flower remain to be elucidated in common buckwheat. Furthermore, *F. esculentum* belongs to family Polygonaceae in the order Caryophyllales, an early diverging core eudicots clade, and produces distylous flowers with undifferentiated petaloid tepals, representing a considerable difference with most core eudicots and major crops flowers, which make it an excellent model for exploring floral organ specification and evolution [7,8,9].

In *Arabidopsis thaliana*, the B-class MADS-box genes *APETALA3* (*AP3*) and *PISTILLATA* (*PI*) work together to control the formation of petals and stamens during *Arabidopsis* flower development [10]. The *AP3*-like and *PI*-like genes originated from a duplication event of ancient class B genes that preceded the origin of extant angiosperms [11]. In addition, another major gene duplication event in the *AP3*/*DEFICIENS* (*DEF*) lineage occurred close to, or at the base of, the core eudicot origination, and resulted in the *euAP3*-like and *TM6*-like genes [12]. Moreover, most *euAP3*-like genes express in petals and stamens and specify petal and stamen development, while most *TM6*-like genes show a broader expression zone in flowers but mainly regulate stamen development [13,14]. Moreover, more and more studies suggested that small-scale gene duplication could also lead to sub- or neo-functionalization of *AP3*-like genes in different taxa [14,15,16].

Here, we found that a small-scale gene duplication (GD) event of buckwheat *AP3* ortholog resulted in *FaesAP3_1* and *FaesAP3_2*. Furthermore, alternative splicing (AS) generates two transcript isoforms from the *FaesAP3_2* after the gene duplication. Both AS isoforms, *FaesAP3_2* and *FaesAP3_2a*, differ by 32 nucleotides in length and result from alternative 5′splice-site selection. Our previous study suggested that *FaesAP3_1* is involved only in stamen development in *F. esculentum* [9]. In the present study, the functional diversification of buckwheat *AP3*-like gene was analyzed by characterizing their promoters. In addition, the function of *FaesAP3_2* and *FaesAP3_2a* specifying floral organ identity were analyzed by assessing their ability to rescue the phenotype of *Arabidopsis ap3-3* mutant. The possible impacts of GD and AS on the function of three buckwheat *AP3*-like genes were investigated, and the functional diversification among them was proposed. Our findings also provide clues for tracing the structure and functional evolution of euAP3-type genes in the early diverging core eudicots.

## 2. Results

### 2.1. Isolation and Characterization of FaesAP3_2 and FaesAP3_2a from F. esculentum

**Figure 2:**
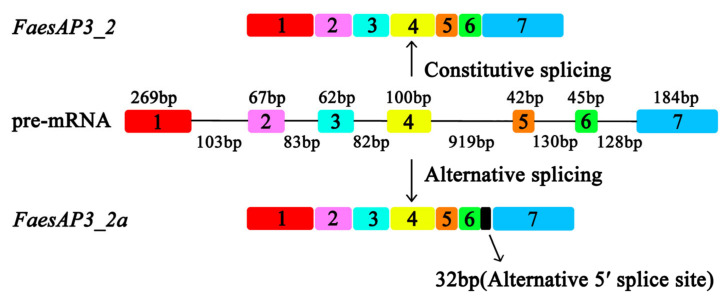
Alternative splicing of *FaesAP3_2.* Color boxes indicate exons while black lines indicate introns of *FaesAP3_2* gene.

**Figure 3:**
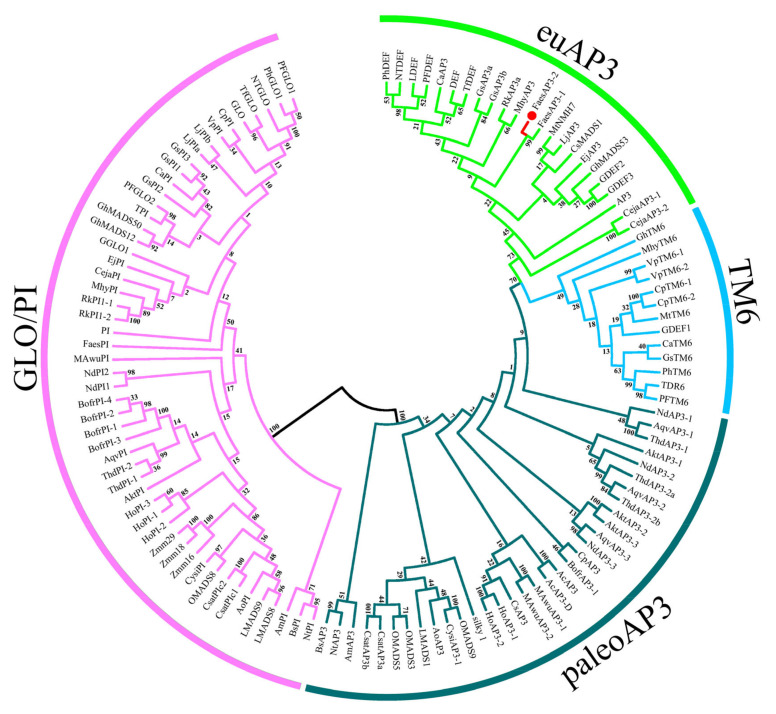
Phylogenetic tree of *FaesAP3_2* and other B-class MADS-box transcription factors from different clades of angiosperms.

**Figure 4:**
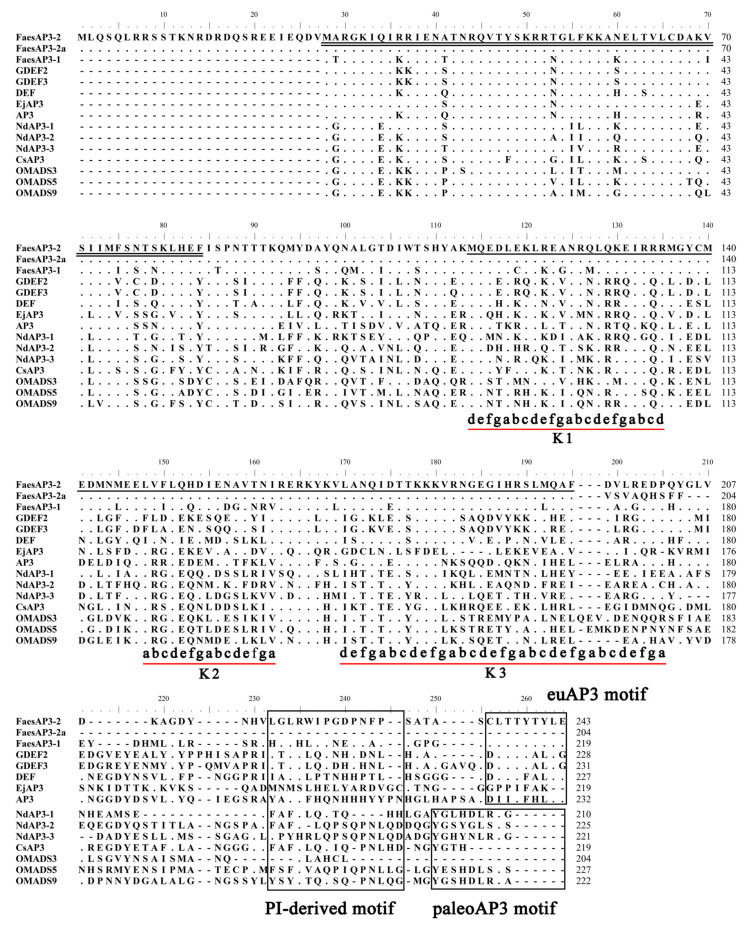
Sequence alignments of *FaesAP3_2* and *FaesAP3_2a* with other *AP3*-like proteins from different clades of angiosperms. The double black underline represents the MADS domain and the single black underline the K domain. The conserved PI-derived motif, euAP3 motif and paleoAP3 motif located at the various C-terminal regions are boxed. The dots represent identical amino acid residues with *FaesAP3_2*. Dashes introduced into the sequence to improve the alignment. Three red underlines represent k1, k2 and k3 subdomains with (abcdefg)n repeats and usually with hydrophobic amino acids at positions a and d [19].

The 891 bp *FaesAP3_2* cDNA contains a 732 bp ORF (Open Reading Frame, ORF) encoding 243 amino acids (aa) (Genbank accession number: MN016949.1), while the ORF of the *FaesAP3_2a* is only 615 bp and encodes 204 aa (Genbank accession number: MN016950.1). Moreover, genomic DNA sequence of *FaesAP3_2* (Genbank accession number: MN016951.1) is 2214 bp consisting of seven exons and six introns. Sequences alignment revealed that *FaesAP3_2* and *FaesAP3_2a* were derived from the consensus pre-mRNA, and both variants differed at the exon 6-intron 6 splice junction sites which resulted in a 32 bp nucleotides addition in the sixth exon of *FaesAP3_2a* from alternative 5′splice-site selection than the sixth exon of the *FaesAP3_2* (Figure 2) [17,18]. Phylogenetic tree analysis grouped *FaesAP3_2* into euAP3 lineage (Figure 3), and the protein sequence also showed 67.89% identity with the *FaesAP3_1* (Genbank accession number: AFO83616.1), another common buckwheat *AP3*-like MADS-box transcription factor. The gene was designated as *FaesAP3_2* (*Fagopyrum esculentum APETALA3_2*). Proteins alignment shows that FaesAP3_2 protein comprises a 27 amino acids (aa) seldom seen N-terminal extension region, a 57 aa highly conserved MADS-box domain (28–84), a 82 aa moderately conserved K domain (114–195) in the middle region and a 48 aa variable C-terminal region (196–243), but with two conserved motifs: PI-derived motif and an euAP3 motif (Figure 3) [12,19]. Moreover, *FaesAP3_2* includes three putative amphipathic α-helices designated as K1 (114–135), K2 (148–162) and K3 (170–205) subdomains containing conserved hydrophobic amino acids residues at the a and d positions in the (abcdefg)n heptad repeats [19]. The 32 bp nucleotides addition in the sixth exon of *FaesAP3_2a* results in frameshift mutation and consequent omission of the complete PI-derived motif and euAP3 motif (Figure 4), two key conserved motifs located at the C-terminal region of the *euAP3*-like transcription factors that are involved in mediating protein–protein interactions [12].

### 2.2. Expression Analysis of FaesAP3_2 and FaesAP3_2a

**Figure 5:**
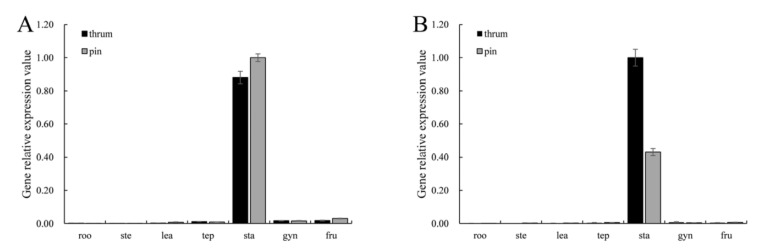
*FaesAP3_2* and *FaesAP3_2a* expression in the roots (roo), stems (ste), juvenile leaves (lea), tepals (tep), stamens (sta), gynoecia (gyn) and 4-day-old fruits (fru) of *F. esculentum* by qRT-PCR with *Faesactin* as the internal control. (**A**) *FaesAP3_2* expression in different tissues of pin and thrum flower plants were detected by qRT-PCR, respectively; (**B**) *FaesAP3_2a* expression in different tissues of pin and thrum flower plants were detected by qRT-PCR, respectively.

**Figure 6:**
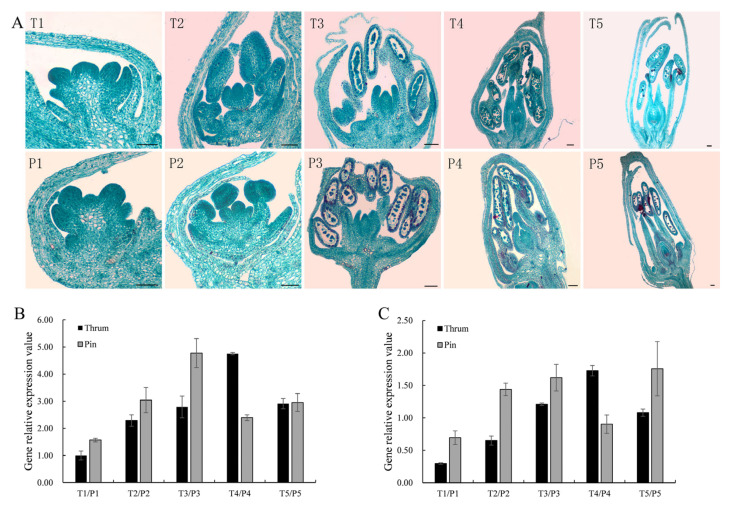
Morphology, *FaesAP3_2* and *FaesAP3_2a* expression in common buckwheat distylous flowers at sequential developmental stages. (**A**) Morphology of thrum and pin flowers at various development stages; T1–T5: morphological differentiation and development of the thrum flower buds; T1: stamen primodium emergence; T2: stamen filament elongation; T3: microspore mother cells meiosis; T4: mononuclear microspore at Periphery, tepal enclosing; T5: full maturity flower buds with mature pollen and embryo sac before anthesis; P1–P5: morphological differentiation and development of the pin flower buds; P1: stamen primodium appearance; P2: stamen filament rapid elongating; P3: microspore mother cells meiosis and formation of microspores tetrads; P4: microspore released from tetrads and tepal enclosing; P5: full maturity flower bud with mature pollen and embryo sac before anthesis; (**B**) *FaesAP3_2* expression at sequential development stages of thrum and pin flower buds were detected by qRT-PCR, respectively; (**C**) *FaesAP3_2a* expression at sequential development stages of thrum and pin flower buds were detected by qRT-PCR, respectively; scale bar: 100 μm.

*FaesAP3_2* was mainly expressed in stamen of thrum and pin flower, but *FaesAP3_2a* transcript was detected only in stamen (Figure 5A,B). Moreover, weak transcripts of *FaesAP3_2* was also observed in tepal, gynoecia and 4-day-old fruit of pin and thrum flowers (Figure 5A). *FaesAP3_2* and *FaesAP3_2a* expressions became apparent after stamen primodium emerge in thrum and pin flower buds (Figure 6A–C). In addition, *FaesAP3_2* expression increased constantly and reached the peak until mononuclear microspore at Periphery formation and tepal enclosing in thrum flower buds (T4), but microspore mother cells meiosis and microspores tetrads formation occurred in the pin flower bud (P3), then *FaesAP3_2* expression began to decline (T5, P4) (Figure 6A,B). In addition, *FaesAP3_2a* expression was similar with the *FaesAP3_2* during the thrum and pin floral bud development (Figure 6A–C). Furthermore, *FaesAP3_2* and *FaesAP3_2*a expression persisted in a relative high level during stamen filament elongation and anther development in pin and thrum flowers (T2–T4, P2–P3).

### 2.3. Isolation and Identification of FaesAP3_1 and FaesAP3_2 Promoters from F. esculentum

A 2.4 kb *pFaesAP3_1* (−2295/+135) (Genbank accession number: MK956946.1) and a 1.5 kb *pFaesAP3_2* (−1401/+122) (Genbank accession number: MN016952.1) were isolated from *F. esculentum*, respectively. The putative transcription start site and cis-acting regulatory elements of both promoters were shown in Figures S1 and S2, respectively (Supplementary Figures S1–S2). Promoter *pFaesAP3_1* contains a key CArG-box motif (−2160/−2151) for MADS-box transcription factor recognition and binding [20], which is also found in the promoter region of *pFaesAP3_2* (−338/−329). In addition, each promoter contains two POLLEN2LELAT52 boxes and four GTGANTG10 boxes, which are essential for stamen-specific gene expression [21,22]. Furthermore, both promoters also contain binding sites (CCAATBOX1) for CONSTANS protein to regulate flowering [23]. Moreover, the MYCCONSENSUSAT box and ACGTATERD1 box are found in *pFaesAP3_1* and *pFaesAP3_2*, which suggests that the corresponding gene expression could be induced by freezing/dehydration stress [24,25]. Some gibberellin-responsive elements, such as WRKY71OS-box, and PYRIMIDINEBOXOSRAMY1A/PYRIMIDINEBOXHVEPB box [26,27], are also found in *pFaesAP3_1/2*, which suggested that the both genes could be regulated by gibberellin-responsive gene. In addition, several mesophyll-specific elements CACTFTPPCA1 boxes are also lying at *pFaesAP3_1* and *pFaesAP3_2* region, which suggested that the corresponding gene expression may extend to leaves [28].

**Figure 7:**
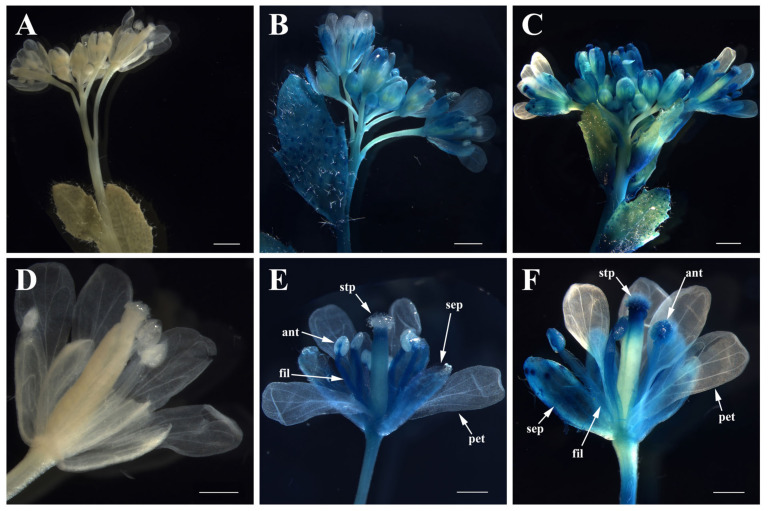
Histochemical GUS staining of *pFaesAP3_1::GUS* and *pFaesAP3_2::GUS* transgenic *Arabidopsis*, respectively. (**A**) Wild-type *Arabidopsis* inflorescence; (**B**) inflorescence of *pFaesAP3_1::GUS* transgenic *Arabidopsis;* (**C**) inflorescence of *pFaesAP3_2::GUS* transgenic *Arabidopsis*; (**D**) wild-type *Arabidopsis* flower; (**E**) flower of *pFaesAP3_1::GUS* transgenic *Arabidopsis*; (**F**) flower of *pFaesAP3_2::GUS* transgenic *Arabidopsis*. Sepal (sep), petal (pet), anther (ant), filament (fil), stigmatic papillae (stp); scale bars: (**A**–**C**) 1 mm; (**D**–**F**) 500 μm.

A *GUS* reporter gene driven by *pFaesAP3_1* or *pFaesAP3_2* was activated in the cauline leaf, inflorescence rachis and flower of transgenic *Arabidopsis* (Figure 7B,C). Furthermore, GUS staining was observed in the flower where sepal, filament and stigma staining were intense, but was almost absent in petal, anther and stigmatic papillae of *pFaesAP3_1*::*GUS* transgenic *Arabidopsis* (Figure 7E). However, GUS staining was observed in the flower where sepal, stamen (filament and anther), stigma and stigmatic papillae staining were intense, but was almost absent in the petal of *pFaesAP3_2*::*GUS* transgenic *Arabidopsis* (Figure 7F).

### 2.4. Ectopic Expression of FaesAP3_2 and FaesAP3_2a in Arabidopsis ap3-3 Mutant

To uncover the roles of *FaesAP3_2* and *FaesAP3_2a* involved in floral development, *35S::FaesAP3_2* and *35S::FaesAP3_2a* constructs have been transformed into heterozygote *AP3/ap3-3 Arabidopsis* to create complementation lines. All transgenic plants were confirmed by PCR and qRT-PCR. Furthermore, the independent transgenic lines of both *35S::FaesAP3_2* and *35S::FaesAP3_2a Arabidopsis* under wild-type, heterozygote and homozygous background were confirmed by dCAPS method with *Cla* I restriction enzymes, respectively (Supplementary Figure S3). In addition, ectopic expression of *FaesAP3_2* or *FaesAP3_2a* in transgenic lines under wild-type and homozygous background were detected, respectively. Moreover, 14 *35S::FaesAP3_2* lines under wild-type background, 21 independent *35S::FaesAP3_2* lines under *AP3/ap3-3* heterozygote background and 8 independent *35S::FaesAP3_2* lines under homozygous *ap3* mutant background were obtained, respectively. In addition, 9 *35S::FaesAP3_2a* transgenic *Arabidopsis* plants under wild-type background, 16 *35S::FaesAP3_2a* transgenic *Arabidopsis* plants under heterozygote background and 10 *35S::FaesAP3_2a* transgenic *Arabidopsis* plants under homozygous background were also obtained, respectively. Phenotypes of different transgenic lines after flowering were assessed to evaluate whether *FaesAP3_2* or *FaesAP3_2a c*ould replace the endogenous *AP3* gene to control petal and stamen development in *Arabidopsis* *ap3-3* mutant.

**Figure 8:**
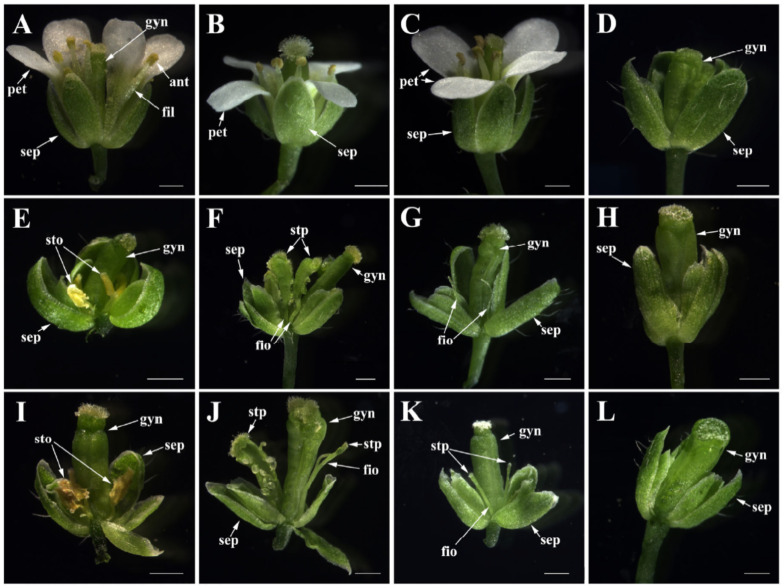
Flower phenotypes comparison of the wild-type, *Arabidopsis ap3-3* mutant, *35S::FaesAP3_2* transgenic *Arabidopsis ap3-3* mutant and *35S::FaesAP3_2a* transgenic *Arabidopsis ap3-3* mutant. (**A**) Wild-type *Arabidopsis* flower with normal flowers (sepal in whorl 1, petal in whorl 2, stamen in whorl 3 and fused carpel in whorl 4); (**B**) flower of *35S::FaesAP3_2* transgenic *Arabidopsis* under wild-type background; (**C**) flower of *35S::FaesAP3_2a* transgenic *Arabidopsis* under wild-type background; (**D**) flower of *Arabidopsis ap3-3* mutant with petal converted into sepal and stamen deficiency in whorl 3; (**E**) flower of *35S::FaesAP3_2* transgenic homozygous *ap3-3* *Arabidopsis* with stamen-like organs in whorl 3; (**F**) flower of *35S::FaesAP3_2* transgenic homozygous *ap3-3 Arabidopsis* with filament attached with a carpelloid anther in whorl 3; (**G**) flower of *35S::FaesAP3_2 t*ransgenic homozygous *ap3-3 Arabidopsis* with 1~2 filament-like organs in the stamen whorl; (**H**) flower of *35S::FaesAP3_2a* transgenic homozygous *ap3-3 Arabidopsis* without phenotype complementation; (**I**) flower of *35S::FaesAP3_2a* transgenic homozygous *ap3-3* *Arabidopsis* with stamen-like organs in whorl 3; (**J**) flower of *35S::FaesAP3_2a* transgenic homozygous *ap3-3* *Arabidopsis* with filament attached with stigmatic papillae or carpelloid anther in whorl 3; (**K**) flower of *35S::FaesAP3_2a* transgenic homozygous *ap3-3* *Arabidopsis* only with filament-like organs or filament attached with stigmatic papillae in whorl 3; (**L**) flower of *35S::FaesAP3_2a* transgenic homozygous *ap3-3* *Arabidopsis* without phenotype complementation. Sepal (sep), petal (pet), anther (ant), filament (fil), filament-like organ (fio), stamen-like organ (sto), gynoecia (gyn), stigmatic papillae (stp); scale bars: 500 μm.

Among eight *35S::FaesAP3_2* transgenic *ap3-3 Arabidopsis*, two (25.00%) showed a strong capability to rescue stamen-like organs in the third whorl of the flower (Figure 8E), one (12.50%) displayed a medium capability to produce filament attached with carpelloid anther in whorl 3 (Figure 8F), two (25.00%) with a weak complement phenotype have flowers with 1–2 filament-like organs in the stamen whorl (Figure 8G) and the remaining three lines (37.5%) displayed no complementation and produced flowers similar to the flowers of *Arabidopsis* *ap3-3* mutant (Figure 8H). Among 10 *35S::FaesAP3_2a* transgenic *Arabidopsis ap3-3* mutants, 3 (30.00%) completed stamen-like organ development in the third whorl of the flower (Figure 8I), 2 (20.00%) with a medium-rescued phenotype produced flowers with filament attached with stigmatic papillae or carpelloid anther in whorl 3 (Figure 8J), two (20.00%) displayed a weak capability to produce flowers only with filament-like organs or filament attached with stigmatic papillae in whorl 3 (Figure 8J) and the other three (30.00%) lines showed no complementation phenotype (Figure 8K). In addition, neither *35S::FaesAP3_2* transgenic lines nor *35S::FaesAP3_2a* transgenic lines under wild-type *Arabidopsis* background displayed flower phenotype change, and all the transgenic lines under wild-type *Arabidopsis* background produce flowers similar to the flowers of wild-type *Arabidopsis* (Figure 8B,C). Moreover, the expression levels of *FaesAP3_2* and *FaesAP3_2a* corresponded to the degree of flower-phenotype complementation in transgenic *ap3-3 Arabidopsis* (Supplementary Figure S4). For example, *FaesAP3_2* expression levels in transgenic line of homozygous *ap3-3 Arabidopsis* with strong complementation phenotype (T_1_-2-8/*ap3-3*) were significantly higher than that of the weak complementation phenotype line (T_1_-2-4/*ap3-3*) and no phenotype complementation line (T_1_-2-2/*ap3-3*) (*LSD*, *p* < 0.05). Similar results were also observed in *35S::FaesAP3_2a* transgenic *ap3-3 Arabidopsis.* For example, *FaesAP3_2a* expression levels in homozygous *ap3-3* transgenic *Arabidopsis* with strong complementation phenotype (T_1_-2a-10/*ap3-3*) were significantly higher than that of the weak complementation phenotype line (T_1_-2a-5/*ap3-3*) and no phenotype complementation line (T_1_-2a-1/*ap3-3*) (*LSD*, *p* < 0.05).

## 3. Discussion

Previous studies suggested that two paralogous lineages (*euAP3* and *TM6*) of *AP3*-like genes in the core eudicots resulted from a duplication event of the ancestral paleoAP3 lineage within the basal eudicots [12]. Following the duplication, the euAP3 lineage acquired a novel C-terminal euAP3 motif instead of paleoAP3 motif and a new role in regulating petal development, while TM6 has preserved the C-terminal paleoAP3 motif [12,29]. In core eudicots, most euAP3-type genes, such as *EjAP3* from *Eriobotrya japonica* [30], *MtNMH7* from *Medicago truncatula* [31], *PFDEF* from *Physalis floridana* [32] and *GDEF2* from *Gerbera hybrid* [33], were expressed only in petal and stamen, and were mainly involved in specifying petal and stamen identities during flower development. All these studies demonstrated that the functions of euAP3-type genes are highly correlated with their expression pattern in core eudicots. However, most *TM6*-like genes usually showed broader expression zones, but were involved only in stamen development in core eudicots. For examples, *Medicago truncatula TM6*-like gene *MtTM6* was expressed predominantly in the outer cell layers of petal and stamen, but played a key role involving in stamen development [31]. *Physalis floridana TM6*-like gene *PFTM6* was expressed in corolla, androecium and gynoecium, but was involved in pollen maturation [32]. *Gerbera* hybrid *TM6*-like *GDEF1* was expressed in all four floral whorls of disk flower, but had a redundant role in determining stamen development [14]. Besides the broader expression patterns, small-scale gene duplication event of the *paleoAP3*-like genes were observed throughout basal eudicots with petaloid sepals and basal angiosperms with undifferentiated perianth (petaloid tepals). For examples, *NdAP3-1*, *NdAP3-2* and *NdAP3-3* were three paleoAP3-type genes found in basal eudicots *Nigella damascene* (Ranunculaceae)*. NdAP3-3* was mainly expressed in petal and specified petal identity, while *NdAP3-1* and *NdAP3-2* have much broader expression domains (sepal, petal, stamen and carpel) and have overlapping functions in specifying stamen identity [33]. Three paleoAP3-type genes, *AqAP3-1*, *AqAP3-2* and *AqAP3-3*, were also found in *Aquilegia coerulea* (Ranunculaceae). *AqAP3-1* and *AqAP3-2* were obviously expressed in sepals, petals, staminodia, stamens and carpels, and work together to specify stamen identity, while *AqAP3-3* was mainly expressed in petals and was required only for petal identity [16]. Two paleoAP3-type genes, *MAwuAP3_1* and *MAwuAP3_2*, were also found in basal angiosperms *Magnolia wufengensis* (magnoliids); both genes were mainly expressed in petaloid tepal and stamen, but were required only for stamen development [34]. All these data suggested that stamen-specific function of *AP3*-like genes antedate their petal-specific identity during angiosperm evolution.

Gene duplication events and AS are often associated with shifts in expression patterns and/or changes in coding sequence, giving rise to the diversification of gene function [12,16,35]. In *F. esculentum*, *FaesAP3_1* was expressed only in stamen and exclusively required for stamen formation [9], while *FaesAP3_2* expression extended to petaloid tepal and gynoecia although the expression was so slow. In addition, GUS staining was observed in the whole stamen of *pFaesAP3_2::GUS* transgenic *Arabidopsis*, while GUS staining was observed only in the filament of *pFaesAP3_1::GUS* transgenic *Arabidopsis*, but was absent in the anther. All these data may suggest that *FaesAP3_1* and *FaesAP3_2* had overlapping functions in specifying stamen filament identity, and *FaesAP3_2* played a key role in regulating anther development. Both genes work together to control normal stamen development. Intense GUS staining was also observed in the stigma and stigmatic papillae of *pFaesAP3_2::GUS* transgenic *Arabidopsis*. Moreover, phenotype complementation analysis suggested that some *35S::FaesAP3_2* transgenic *Arabidopsis ap3-3* mutants could produce flowers with a filament attached with stigmatic papillae in whorl 3. These data may suggest that *FaesAP3_2* may be involved in style development. However, the AS isoform *FaesAP3_2a* was expressed only in the stamen of thrum and pin flowers. Phenotype complementation analysis suggested that *FaesAP3_2a* holds a similar ability with *FaesAP3_2* to rescue stamen development of *Arabidopsis ap3-3* mutant even without the C-terminal euAP3 motif, which suggested that both AS isoforms had overlapping functions in specifying stamen identity of common buckwheat though they showed different expression patterns. As most transcriptional factors are modular proteins with multiple functional modules, some truncated protein isoforms containing functional modules may still have function and act as dominant-negative regulators [36]. The *AP3*-like and *PI*-like transcriptional factors are closely related MADS domain proteins that are thought to act as obligate heterodimers, and their I and K domains were required for dimerization and protein stability [10,19]. Hence, truncated *FaesAP3_2a* isoform might still participate in dimerization, which can compete with *FaesAP3_2*. A future challenge remains in exploring how the both AS isoforms work together with other common buckwheat B genes to specify stamen identity. Moreover, previous studies also suggested that the petaloid tepal of common buckwheat were homologs to core eudicots sepal and showed a relative original trait of flowers [7,8]. All these data suggested that the MIK region of *AP3*-like proteins was crucial and essential for determining stamen identity, while the function of *AP3*-like proteins in specifying petal identity was gradually obtained accompanying sepal and petal differentiation after the AP3 orthologs acquired a novel C-terminal euAP3 motif during the evolution of core eudicots. Our data suggested early evolution of the functional specificity of euAP3-type proteins in floral organ development in core eudicots, and also provide an idea candidate gene for biotechnical engineering to develop a sterile male line of *F. esculentum*.

## 4. Materials and Methods

### 4.1. Plant Material

Thrum and pin floral buds at sequential developmental stages were sampled from common buckwheat ‘Beizaosheng’ growing under natural conditions in Jingzhou, China, respectively. In addition, each sample was divided into two aliquots; one was immediately frozen in liquid nitrogen, and then stored at −80 °C until used; another was fixed in FAA (38% formaldehyde: acetic acid: 70% ethanol = 1:1:18, by volume). The root, stem, juvenile leaf, tepal, stamen, gynoecium and 4-day-old fruit of thrum and pin plants were dissected, respectively, immediately frozen in liquid nitrogen and then stored at −80 °C until used. The *Arabidopsis ap3-3* mutant line (CS3086, Landsberg ecotype) seeds were obtained from the ABRC (*Arabidopsis* Biological Resource Center, ABRC) at Ohio State University, Columbus, OH, USA.

### 4.2. Isolation and Characterization of FaesAP3_2 and Its AS Isoform FaesAP3_2a from F. esculentum

Total RNA from common buckwheat floral buds and the first-strand cDNA of 3′ RACE was prepared according to Fang et al. [9]. The 3′ end and 5′ partial cDNA sequences of two buckwheat *FaesAP3_2* isoforms (*FaesAP3_2* and *FaesAP3_2a)* were obtained by using the 3′-full RACE Core Set Ver. 2.0 kit (TaKaRa, Shiga, Japan) with gene-specific primer GSPAP3 based on the manufacturer’s protocol (Supplementary Table S1). The gene-specific primer GSPAP3 was designed based on F01.PB7802 (putative *AP3*-like MADS-box transcription factor gene) of the BioProject ID PRJNA517031 deposited in the NCBI. Common buckwheat genomic DNA was extracted from leaves by using the CTAB Plant Genomic DNA Rapid Extraction Kit (Aidlab, Beijing, China) following the manufacturer’s protocol. The full length of genomic DNA sequence of *FaesAP3_2* was isolated from common buckwheat genomic DNA with the forward primer *DFaesAP3_2F* and the reverse primer *DFaesAP3_2R*. The PCR amplification of *FaesAP3_2* genomic DNA was performed in a 25 µL reaction volume containing 0.5 µL Phanta Max Super Fidelity DNA Polymerase (Vazyme, Nanjing, China). PCR was performed with a 3 min 94 °C denaturation step, followed by 30 cycles of 30 s at 94 °C, 30 s annealing at 58 °C, a 90 s extension at 72 °C, with a final extension period of 10 min. Sequence alignments and phylogenetic analysis of *FaesAP3_2* were referenced to the method described by Liu et al. [37]. Putative *FaesAP3_2* and *FaesAP3_2a* protein sequences, as well as B-class MADS-box transcription factors from different species, were selected for Phylogenetic trees from NCBI Genbank (Supplementary Table S2).

### 4.3. Cytomorphological Examination and Expression Analysis of FaesAP3_2 and FaesAP3_2a

The thrum and pin floral buds of *F. esculentum* fixed in FAA above were dehydrated in a graded ethanol series, cleared in a xylene series, infiltrated with molten paraffin, embedded into paraffin block, serially sectioned and then sections were stained according to Liu et al. [37]. The sections were observed under a CAIKON RCK-40C microscope and subsequently taken photomicrographs.

Total RNA of each sample was prepared for quantitative real-time PCR (qRT-PCR) by using the EASYspin plant RNA Rapid Extraction Kit (Aidlab, Beijing, China) following the manufacturer’s protocol. The first-strand cDNA was synthesized by using the HiScript^®^ II Q RT SuperMix for qPCR kit (Vazyme, Nanjing, China) according to the manufacturer’s protocol. *FaesAP3_2* and *FaesAP3_2a* expressions were detected in root, stem, juvenile leaf, tepal, stamen, gynoecium and 4-day-old fruit of thrum and pin plants by using qRT-PCR according to Liu et al. [8], but with the gene-specific forward primer qFaesAP3_2F and the gene-specific reverse primer qFaesAP3_2R for *FaesAP3_2*, and the gene-specific forward primer qFaesAP3_2aF and the gene-specific reverse primer qFaesAP3_2aR for *FaesAP3_2a*, respectively. Moreover, the expressions of *FaesAP3_2* and *FaesAP3_2a* were also detected in different development stage floral buds of thrum and pin buckwheat through qRT-PCR suggested above, respectively. For qRT-PCR analysis, the reaction was performed on the Line-Gene 9600 Plus Real-time PCR Detection System by using 2 × ChamQ SYBR qPCR Master Mix (Vazyme, Nanjing, China). Amplification fragment of *F. esculentum actin* gene (Genbank accession number: HQ398855.1) was used as the internal control with the forward primers qFaesactinF and the reverse qFaesactinR. qRT-PCR was carried out with three biological replicates, the PCR program was cycled and relative expression levels were calculated according to Liu et al. [8] but with 30 s annealing at 57 °C.

### 4.4. Isolation and Identification of FaesAP3_1 and FaesAP3_2 Promoter from F. esculentum

The *FaesAP3_1* 5′ flanking region was isolated according to Liu et al. [8], but with the gene-specific primer D1pAP3_1SP1, D1pAP3_1SP2 and D1pAP3_1SP3 for the first walking sequencing, and with the gene-specific primer D2pAP3_1SP1, D2pAP3_1SP2 and D2pAP3_1SP3 for the second walking sequencing. In addition, The *FaesAP3_2* 5′ flanking region was isolated according to the method suggested above but with the gene-specific primer FLpAP3_2SP1, FLpAP3_2SP2 and FLpAP3_2SP2 for the walking sequences. Moreover, the putative transcription start site of *FaesAP3_1* was found based on the 5′RACE according to Fang et al. [9]. The putative transcription start site of *FaesAP3_2* was found based on the 5′RACE using the 5′RACE System for Rapid Amplification of cDNA Ends (Invitrogen, Carlsbad, CA, USA) following the manufacturer’s protocol and the gene-specific primer 5RAP3GSP1, 5RAP3GSP2 and 5RAP3GSP3. The cis-acting elements lying at the *pFaesAP3_1* and *pFaesAP3_2* regions were searched in the PLACE database, respectively [38].

The 1.5 kb 5′ flanking region upstream of *FaesAP3_2* translation start was cloned into pCAMBIA1300 vector with the forward primer TpFaesAP3_2F and the reverse primer TpFaesAP3_2R, and restriction enzymes *Xba* I and *Sac* I. *pFaesAP3_2::GUS* construct was transformed into *A. thaliana* *Col-0* plants (ecotype Columbia) using the floral-dip method described by Clough and Bent [39]. Transgenic *Arabidopsis* seedlings were selected, cultivated and prepared for histochemical GUS staining according to Liu et al. [8].

### 4.5. Ectopic Expression Analysis of FaesAP3_2 and FaesAP3_2a in Arabidopsis ap3-3 Mutant

Full-length *FaesAP3_2* and *FaesAP3_2a* cDNAs in the sense orientation were separately cloned into pBI121 vector with *Xba* I and *Sac* I restriction enzymes, and the forward primer TFaesAP3_2/2aF and the reverse primer TFaesAP3_2/2aR under control of the CaMV35S promoter using the ClonExpress^®^ Ultra One Step Cloning Kit (Vazyme, Nanjing, China) according to the manufacturer’s protocol. The *35S::FaesAP3_2* and *35S::FaesAP3_2a* constructs were transformed into *heterozygote* *Ap3/ap3-3 Arabidopsis* using the floral-dip method described by Clough and Bent, respectively [39]. Transgenic *Arabidopsis* seeds were selected, and seedlings were cultivated according to Fang et al. [9]. Homozygous *ap3-3* transgenic *Arabidopsis* lines were identified by dCAPS genotyping following the method suggested by Lamb and Irish [29]. The phenotypes of transgenic *Arabidopsis* were analyzed after flowering. In addition, the complementation degrees of independent transgenic lines of *35S::FaesAP3_2* and *35S::FaesAP3_2a* homozygous *ap3-3 Arabidopsis* were categorized as ‘no complementation’, ‘weak complementation’, ‘medium complementation’ and ‘strong complementation’, respectively. Moreover, independent transgenic lines of each complementation degree were confirmed by qRT-PCR with the primers qTFaesAP3_2F and qTFaesAP3_2R for *FaesAP3_2*, and with the primers qFaesAP3_2a and qFaesAP3_2aR suggested above for *FaesAP3_2a,* respectively. Amplification fragment of *A. thaliana ubiquitin 5* (Genbank accession number: NM_116090.3) with the primers qUBQ5F and qUBQ5R as the internal control.

### 4.6. Statistical Treatment

All experiments were carried out with three biological replicates, and data were expressed as mean ± SE (standard errors). Statistical significance was determined by one-way ANOVA followed by *LSD*, and statistical significance was declared at *p*-value ≤ 0.01 or 0.05 level, respectively.

## 5. Conclusions

Combined with our previous studies, we found that small-scale gene duplication (GD) event and alternative splicing (AS) of buckwheat *AP3* orthologs resulted in *FaesAP3_1, FaesAP3_2* and *FaesAP3_2a*. *FaesAP3_2* and *FaesAP3_2a* were mainly expressed in the stamen of thrum and pin flowers. Promoters functional analysis suggested that intense GUS staining was observed in the whole stamen of *pFaesAP3_2::GUS* transgenic *Arabidopsis,* while intense GUS staining was observed only in the filament of *pFaesAP3_1::GUS* transgenic *Arabidopsis.* These suggested that *FaesAP3_1* and *FaesAP3_2* had overlapping functions in specifying stamen filament identity and *FaesAP3_2* played a key role in regulating anther development. Both genes work together to determine normal stamen development. Additionally, *FaesAP3_2* and *FaesAP3_2a* owned the similar ability to rescue stamen development in *Arabidopsis ap3-3* mutant, although AS resulted in frameshift mutation and consequent omission of the complete PI-derived motif and euAP3 motif of FaesAP3_2a. In addition, previous studies also suggested that the petaloid tepal of common buckwheat were homologs to core eudicots sepal. All these suggested that the MIK region of *AP3*-like proteins was crucial for determining stamen identity, while the function of *AP3*-like proteins in specifying petal identity was gradually obtained after the AP3 orthologs acquired a novel C-terminal euAP3 motif during the evolution of core eudicots. Our results also provide a clue to understanding the early evolution of the functional specificity of euAP3-type proteins in floral organ development in core eudicots, and also suggest that *FaesAP3_2* holds the potential application for biotechnical engineering to develop a sterile male line of *F. esculentum*.

## Data Availability

All data generated or analyzed during this study are included in this published article. Further inquiries can be addressed to the corresponding author.

## Supplementary Materials

The following are available online at www.mdpi.com/article/10.3390/plants10081644/s1, Table S1: Primers used in this study. Table S2: Information on Sequences selected for alignments and phylogenetic analyses from NCBI GenBank. Figure S1: *FaesAP3_1* promoter sequence. Figure S2: *FaesAP3_2* promoter sequence. Figure S3: Genotyping of wild-type, heterozygous and homozygous ap3-3 mutant *A. thaliana* by dCAPS. Figure S4: Expression of *FaesAP3_2* and *FaesAP3_2a* in transgenic *Arabidopsis ap3-3* mutant confirmed by qRT-PCR.
