# Supplementary material for: Isolation and Characterization of APETALA3 Orthologs and Promoters from the Distylous Fagopyrum esculentum
Source: Plants (Basel). 2021 Aug 10;10(8):1644. doi: 10.3390/plants10081644 (PMC8402184; doi:10.3390/plants10081644)
Supplement: Supplementary file 1 [file plants-10-01644-s001.zip › plants-1327084-supplementary.pdf]

Supplementary Materials

Table **S1.** Primers used in this study.

Primer name	Primers sequences (5'-3')
GSPAP3	GAAATAGAACAAGACGTGATGGC
DFaesAP3_2F	ATGGCGAGGGGAAAGATCCAGATAC
DFaesAP3_2R	GTGAGGAACCATATACAATCGGAGAC
qFaesAP3_2F	TGATGTGTTGAGGGAGGATC
qFaesAP3_2R	AATCGGAGACACGAACAGCT
qFaesAP3_2aF	CGTGGCACAGCATTCCTTTT
qFaesAP3_2aR	GAGACACGAACAGCTGCTAC
qFaesactinF	ACCTTGCTGGACGTGACCTTAC
qFaesactinR	CCATCAGGAAGCTCATAGTTC
D1pAP3_1SP1	TCCTCTTGCATCTTCTGTACATCGTC
D1pAP3_1SP2	GGAGTCGTACATCTGCTTGGTGCTAC
D1pAP3_1SP3	GTGGTGTTGGGAGTGATGAATTCGTG
D2pAP3_1SP1	GCGTTGATCGTCCCATTGTTTATTC
D2pAP3_1SP2	TCTTCTGTAATCCATTTGTCTTGCC
D2pAP3_1SP3	CATCCACAACCACCATTTAAGTCCT
FLpAP3_2SP1	AGGATTACGTGGTGTTGGGACTGATG
FLpAP3_2SP2	ATCGCAGAGAACCGTGAGCTCAT
FLpAP3_2SP3	CTTGCCTGTTGGTTGCGTTCTCTATCC
5RAP3_2GSP1	GTGAGGAACCATATACAATC
5RAP3_2GSP2	TGGTATCCAGCGCAGTCCTAGAAC
5RAP3_2GSP3	GTATCAATCTGATTCGCGAGTAC
TpFaesAP3_1F	GAGCTCCGATACGACTCCTACTACGGTTC
TpFaesAP3_1R	TCTAGATTTTGTTACTTGTTGTCCTTAGCAATC
TpFaesAP3_2F	ATGATTACGAATTCGAGCTCAGTTAAACCAGACACATTTACTCCC
TpFaesAP3_2R	TCTGCAGGTCGACTCTAGAAACAAAGTAGGAAGAAAAACTAGGTGGAG
TFaesAP3_2/2aF	AACACGGGGGACTCTAGACCACCTCTTACTCTCTCCACCTAG
TFaesAP3_2/2aR	TCGGGGAAATTCGAGCTCTCCTAAGTAGGAACACATGCAGC
qTFaesAP3_2F	GATTTGGCATCACACTTTCTACAATG
qTFaesAP3_2/R	GTATCCAGCGCAGTCCT
qUBQ5F	AAGACTTACACCAAGCCGAAGAAGATC
qUBQ5R	CCAGCTCCACAGGTTGCGTTAG

Table **S2.** Information on Sequences selected for alignments and phylogenetic analyses from NCBI GenBank.

Taxon and species	Protein name	Accession numbers	Lineage
Amborellaceae			
Amborella trichopoda	AmAP3	BAD42444.1	AP3
	AmPI	BAD42443.1	PI
Nymphaeaceae			
Nymphaea tetragona	NtAP3	BAD42348.1	AP3
	NtPI	BAD42349.1	PI
Cabombaceae			
Brasenia schreberi	BsAP3	BAD42352.1	AP3
	BsPI	BAD42353.1	PI
Aristolochiaceae			
Asarum caudigerum	AcAP3	ACA62949.1	AP3
	AcAP3-D	AGO59776.1	AP3
Magnoliaceae			
Magnolia wufengensis	MAwuAP3-1	AFM75880.1	AP3
	MAwuAP3-2	AFM75881.1	AP3
	MAwuPI	AFM75882.1	PI
Calycanthaceae			

Chimonanthus praecox	CpAP3	ABK34952.1	AP3
Chloranthaceae			
Hedyosmum orientale	HoAP3-1	AFP17801.1	AP3
	HoAP3-2	AFP17802.1	AP3
	HoPI-1	AFP17796.1	PI
	HoPI-2	AFP17799.1	PI
	HoPI-3	AFP17800.1	PI
Chloranthus spicatus	CsAP3	AAR06664.1	AP3
Liliaceae			
Lilium longiflorum	LMADS1	AAM27456.1	AP3
	LMADS8	AEI88009.1	PI
	LMADS9	AEI88010.1	PI
Orchidaceae			
Oncidium hybrid cultivar	OMADS3	AAO45824.1	AP3
	OMADS5	ADJ67234.1	AP3
	OMADS9	ADJ67235.1	AP3
	OMADS8	ADJ67236.1	PI
Cymbidium sinense	CysiAP3-1	AUO28994.1	AP3
	CysiPI	AUO28996.1	PI
Iridaceae			
Crocus sativus	CsatAP3a	AAY24691.1	AP3
	CsatAP3b	AAY24692.1	AP3
	CsatPIc1	ABB22780.1	PI
	CsatPIc2	ABB22781.1	PI
Zingiberaceae			
Alpinia oblongifolia	AoAP3	ABS83561.1	AP3
	AoPI	ABB92623.1	PI
Poaceae			
Zea mays	silky 1	NP_001104951.1	AP3
	Zmm16	NP_001105136.1	PI
	Zmm18	CAC33849.1	PI
	Zmm29	CAC33850.1	PI
Papaveraceae			
Bocconia frutescens	BofrAP3-1	AOC50670.1	AP3
	BofrPI-1	AOC50677.1	PI
	BofrPI-2	AOC50678.1	PI
	BofrPI-3	AOC50679.1	PI
	BofrPI-4	AOC50680.1	PI
Lardizabalaceae			
Akebia trifoliata	AktAP3-1	AAT46097.1	AP3
	AktAP3-2	AAT46098.1	AP3
	AktAP3-3	ABC02398.1	AP3
	AktPI	AAT46101.1	PI
Ranunculaceae			
Aquilegia vulgaris	AqvAP3-1	ABP01804.1	AP3
	AqvAP3-2	ABP01803.1	AP3
	AqvAP3-3	ABP01802.1	AP3
	AqvPI	ABP01801.1	PI
Thalictrum dioicum	ThdAP3-1	AAW78033.1	AP3
	ThdAP3-2a	AAW78034.1	AP3
	ThdAP3-2b	AAW78035.1	AP3
	ThdPI-1	AAW78031.1	PI

	ThdPI-2	AAW78032.1	PI
Nigella damascena	NdAP3-1	ALM95512.1	AP3
	NdAP3-2	ALM95513.1	AP3
	NdAP3-3	AGH39931.1	AP3
	NdPI1	ALM95515.1	PI
	NdPI2	ALM95516.1	PI
Cercidiphyllaceae			
Cercidiphyllum japonicum	CejaAP3-1	ASY97764.1	AP3
	CejaAP3-2	ASY97765.1	AP3
	CejaPI	ASY97767.1	PI
Violaceae Batsch			
Viola yedoensis Makino	VpTM6-1	APQ46143.1	AP3
	VpTM6-2	APQ46144.1	AP3
	VpPI	APQ46145.1	PI
Cucurbitaceae			
Cucumis sativus L.	CsMADS1	AAX37273.1	AP3
Fabaceae			
Medicago truncatula	MtNMH7	AEW43601.1	AP3
	MtTM6	AEW43602.1	TM6
Lotus japonicus	LjAP3	AAX13301.1	AP3
	LjPIa	AAX13299.1	PI
	LjPIb	AAX13300.1	PI
Rosaceae			
Eriobotrya japonica	EjAP3	QGR27127.1	AP3
	EjPI	QGR27129.1	PI
Malvaceae			
Gossypium hirsutum	GhMADS53	AGW23355.1	AP3
	GhTM6	ADX60056.1	TM6
	GhMADS12	ACJ26767.1	PI
	GhMADS50	AGW23352.1	PI
Caricaceae			
carica papaya	CpTM6-1	ABQ51321.1	TM6
	CpTM6-2	ABQ51322.1	TM6
	CpPI	ABQ51323.1	PI
Brassicaceae			
Arabidopsis thaliana	AP3	AAD51903.1	AP3
	PI	BAA06465.1	PI
Polygonaceae			
Fagopyrum esculentum	FaesAP3-1	AFO83616.1	AP3
	FaesAP3-2	QLJ83023.1	AP3
	FaesPI	AFO83617.1	PI
Ericaceae			
Rhododendron kaempferi	RkAP3a	BBC77405.1	AP3
	RkPI1-1	BBA27230.1	PI
	RkPI1-2	BBA27231.1	PI
Pyrolaceae			
Monotropa hypopitys	MhyAP3	AQM52302.1	AP3
	MhyTM6	AQM52303.1	TM6
	MhyPI	AQM52304.1	PI
Rubiaceae			
Coffea arabica	CaAP3	AHW58039.1	AP3
	CaTM6	AHW58030.1	TM6

	CaPI	AHW58035.1	PI
Gentianaceae			
Gentiana scabra Bunge	GsAP3a	BAS04474.1	AP3
	GsAP3b	BAS04479.1	AP3
	GsTM6	BAS04472.1	TM6
	GsPI1	BAS04475.1	PI
	GsPI2	BAS04476.1	PI
	GsPI3	BAS04478.1	PI
Solanaceae			
Petunia x hybrida	PhDEF	AAQ72510.2	AP3
	PhTM6	AAS46017.1	TM6
	PhGLO1	AAS46018.1	GLO
Solanum lycopersicum	LDEF	NP_001234077.2	AP3
	TDR6	NP_001311309.1	TM6
	TPI	ABG73411.1	PI
Physalis pubescens	PFDEF	AGN54423.1	AP3
	PFTM6	AGN54424.1	TM6
	PFGLO1	AGN54427.1	PI
	PFGLO2	AGN54426.1	PI
Nicotiana tabacum	NTDEF	CAA65288.1	AP3
	NTGLO	CAA48142.1	GLO
Scrophulariaceae			
Antirrhinum majus L	DEF	BAI68389.1	AP3
	GLO	CAA48725.1	GLO
Torenia fournieri	TfDEF	BAG24492.1	AP3
	TfGLO	BAJ15423.1	GLO
Asteraceae			
Gerbera hybrid	GDEF2	CAA08803.1	AP3
	GDEF3	ACV53813.1	AP3
	GDEF1	CAA08802.1	TM6
	GGLO1	CAA08804.1	GLO

> *pFaesAP3_1*

−2295 CGATACGACT CC**TACT**ACGG TTC**GTGA**CCT CCATGCTTCT GAACTGATCA TGTTTACGAT

CACTFTPPCA1 GTGANTG10

−2235 TATGTAAAAA ACATAAT**CAA CTC**CACCGTG GTGGGGTTAG TCTTCC**ACGT** AGACTAAG**CC**

CAREOSREP1 ACGTATERD1

−2175 **AAT**ACATAAG CGATG**CATAT ATATG**A**ACGT G**ACATGAGTA TAAGCATAAA TAATAT**CATA**

CCAATBOX1 CArG-box ACGTATERD1

−2115 **TG**ATCTTTGG TAA**TACT**CAA AGGATTAAAT ATGCAACATG CTTTCAAAAT CATTATAAGT

MYCCONSENSUSAT CACTFTPPCA1

−2055 CTCAAAATGT TCAAATCATC ATGTCT**CAAT** ACTTAAAATA TCTCAAAAGG TTTCAAACCG

CAATBOX1

−1995 ATATCATCTC AAAAGTA**TAC T**CA**TAACCA**T **CAAGTG**TTTA AAAT**CAAT**AT CATCTCGATA

CACTFTPPCA1 MYB1AT MYCCONSENSUSAT CAATBOX1

−1935 ACTCGTAAAC ATCTCAAAAG AGTTCAAAGT **CAAT**CTCATC T**CAAT**AACTC GTAATCCAAA

CAATBOX1 CAATBOX1

−1875 TCTAGTAACA CATCAAAAT**C AAT**CTCATCT **CAAT**AACTCG TAATCCAAAT CATGTAACTC

CAATBOX1 CAATBOX1

−1815 AT**CAAT**ATCA TCT**CAAT**AAC TCGTAATCCA AATTTATCAG TATAACATAT CTAATGGTCA

CAATBOX1 CAATBOX1

−1755 ATTGA**CCGAA A**TGGGGTAAC AAGGTCGATC CTTGCCCCTT TCTCAAATCC ATCATAAAAA

LTRE1HVBLT49

−1695 TCATCCAAAT CA**CAAT**ACAA AATATGCTCG AGCATTTT**TA CT**T**TATAAAT** TGTATT**CAAC**

CAATBOX1 CACTFTPPCA1 TATABOX2

−1635 **TC**ACGTGTTT GTATT**TTATT T**TAAAACATG TCCAACCCAT GTTGAACACG ATGATACCTC

CAREOSREP1 TATABOX5

−1575 CTAACCT**TGA C**CATGAGATT CTCACAG**CAA T**CACGGAT**CA AGTG**CAGTCC TCGTCATCCA

WRKY71OS CAATBOX1 MYCCONSENSUSAT

−1515 **CAAT**GACCGG GCACAAACTC GGCCCACACA AACTAAGATT ACAAC**CACT**A **GTGA**GCATGA

CAATBOX1 CACTFTPPCA1 GTGANTG10

−1455 CCCGCCTAAA CCGAGGAGCA TAAAAATCAT GAAAACAAAA CGGATGGAAA A**TACT**TCGGT

CACTFTPPCA1

−1395 GTGGTCATAG AC**CAACTC**TT CTCT**CAAT**AT AATAGGGCAA ATTGTCCGCT ATTGTCGTAG

CAREOSREP1 CAATBOX1

−1335 ACCAAATTAT CTCTTG**TGTC ACA**TA**AGAAA** AAAATGCCGC TTAAGTCAAA GACCAAATCA

TGTCACACMCUCUMISIN POLLEN1LELAT52

−1275 TCTCAAATCC T**CAAT**AT**CAA T**AACC**CACT**T CTTTTCCAAA ATATTT**CAAT** AAAATTTCTT

CAATBOX1 CAATBOX1 CACTFTPPCA1 CAATBOX1

−1215 TAATGATCAC AATCAAAATT AA**CACT**TCAC CG**CCAAT**ATT GTCAAATCAA CACAAGGCAA

CACTFTPPCA1 CCAATBOX1

−1155 GTATTCTCAA AGGCTTTTA**C AAT**TGACATA TCAAA**CTCAT TTC**ATATTCT C**AAACCA**AAG

CAATBOX1 INRNTPSADB MYB1AT

−1095 A**CAAT**ATCAT TA**CACT**TTCC ATAAACTTT**C AAT**AACACTT ATCTTTAAAC ATAACAGCGT

CAATBOX1 CACTFTPPCA1 CAATBOX1

−1035 ATAAAGCATT ACAACTAAAG TGTTA**TGAAA AGT**CA**TGAC**A AAATCAAATA ATATATGGGT

-300ELEMENT WRKY71OS

−975 TAAAATAGTA GGGGGAAAAT GCCTAAAATG **TACT**TGTAT**G TGA**AAATA**CA AATG**TGGAAA

CACTFTPPCA1 GTGANTG10 MYCCONSENSUSAT

−915 ACATATTATG GTAATGGAAA AAACTAATTA AACTCAGAGA AGATTTCGAA AAAAGGACTT

−855 AAATGGTGGT TGTGGATGGT A**AAACCA**TTA ACTTCTGGCA AGA**CAAATG**G ATTACAGAAG

MYB1AT MYCCONSENSUSAT

−795 AACCT**CAAT**A AAAATTTGAT CAACTCTACA AAACTAATTA CCAACTACAT CATGAATAAA

CAATBOX1

−735 **CAAT**GGGACG ATCAACGCAT TGAT**CACT**AC ATA**CACT**TTT ACCGATTGAT ATTGTTA**AGA**

CAATBOX1 CACTFTPPCA1 CACTFTPPCA1

−675 **AA**ATTAAAAA ACATAACCTT CCCATGAAGG AGATAAATGA TAGAATATAA TGGGCAA**CAA**

POLLEN1LELAT52

−615 **ATG**AGGAAGA GTGG**TACT**TA TCTTAGTGGC GGAAGCCTTC GCTCTTCGGG AGGGTCTACA

MYCCONSENSUSAT CACTFTPPCA1

−555 AAAGGCCGCT AGCATTGATG CCACATCCAT TGAGATTACA GGAGACAACT TAATATTCAT

−495 GAACTCGGCC AACCAAATTC AGAAGCCTCC ATGCTAGACA AACA**TGAC**CA ACTCAAATAT

WRKY71OS

−435 TTCCA**CAAT**G ATCCCGGGTT TTATGCATGA TA**ACGT**GCAA CACATTTCCG GGGAAATCAA

CAATBOX1 ACGTATERD1

−375 CA**CGGTTG**CT GATCGACTCG CGTTACATGA ACA**CAAT**CCG AATGT**TGAC**G ACGTTATCAT

MYBCORE CAATBOX1 WRKY71OS

−315 TT**CACATG**AT GTTGATGACC TCAT**CCGAAA** AAATGCTATA GGACCTA**CAA T**TT**TGTAAAA**

MYCCONSENSUSAT LTRE1HVBLT49 CAATBOX1

-300ELEMENT

−255 **T**TGACCGTTT ACTCCTTACC TTGGTCAAAA AAATAAAATA AATAAAAATG T**CATGTG**TAA

MYCCONSENSUSAT

−195 ACT**TACT**GCT **G**CGACATTAT CTAAGCAAAA TATCAGTTTT CCGT**CACT**GT CTGCCGTTTG

CACTFTPPCA1 CACTFTPPCA1

−135 ACCGCGA**AAA CCA**TCAAACG GCGTTCCGT**C CAAT**CAATCA AAGCGCTATT GACGACTTC**C**

MYB1AT CCAATBOX1

−75 **ACT**CTTTACA CAGTTAATTG ACGGAAAGTA AATGTGGGAG GGGTTTAAAT ATGGGACTCT

CACTFTPPCA1 +1

−15 G**CAAT**TGAAA CTTTC**A**T**CAA T**GCTTCGCAA GGC**TTATTT**T TCCATTTGTA GTAACCTCTT

CAATBOX1 CAATBOX1 TATABOX5

+46 **CCTTTT**TCTC TCGTTGGAAC TTCAAAG**GTG A**TCTTGAGTT CAAGCCAAGG GAAGAACTAG

PYRIMIDINEBOXOSRAMY1A GTGANTG10

+106 AGAGATTGCT AAGGACAACA AGTAACAAAA **ATG**

GAREAT

Figure S1: ***FaesAP3_1* promoter sequence.**

The transcription start site (+1) is in bold and boxed. The start codon ATG is in bold and boxed. Putative cis-acting regulatory elements are in bold and underlined.

> *pFaesAP3_2*

−1401 CAGTTAAACC AGACACATT**T ACT**CCCAAAT TTTTAT**CAAA TG**CCAGCTTC TGAATGGTTT

CACTFTPPCA1 MYCCONSENSUSAT

−1341 ATGAAGAACA T**TAACTG**CAA TATCGCCT**CA AT**TAAGGGAC TTCCATGGAA CACGATATTT

MYB2AT CAATBOX1

−1281 ACGTACT**CAA T**CTGGTGGTT GTGGAAGTGG CGGTGTAACC GTA**CAAT**GGG GAAAGACAAT

CAATBOX1 CAATBOX1

−1221 ACTCCTCCCC AGAAGAGTGT AT**TTATCCTC AAT**CAAGCCT CTGAA**CATAT G**AATG**TTATT**

SREATMSD CAATBOX1 MYCCONSENSUSAT TATABOX5

−1161 **T**CGACGGCAA GGAGACTGGG GACT**CAAT**CC CGGAAAGAAG AGGTTTGTAT **CAGCTG**GAGG

CAATBOX1 MYCCONSENSUSAT

−1101 AGACCACCTT CTGGTTGGGT TAAGTGGAAT TC**TGAC**GGAG CTGTCAAACA AGGCTGCGGG

WRKY71OS

−1041 GATGCAGCGG CAGGAGGCAT CCTC**AGAAA**C GACGAAGGGC AGTGGCTGCA AGGGAGAGTC
POLLEN1LELAT52

−981 CACCACATTG AA**CAAT**GTTC AGTACCAGCA GCAGAATTGT GGGGTCTTC**T ACT**TGGATTG
CAATBOX1 CACTFTPPCA1

−921 GAAATGTGTT GGGAGCTATG ATTCAGGAAA GTGGAGTTTG AAACAGATTC AAAGCTCATA

−861 GTACAACTAA T**TGAAAAGG**G AACAGCCCAT GCTCACCCCC TTGCTAGCCT GGTGGGTTGC
-300ELEMENT

−801 TGCCAAGGCT TCATACGTAG GAATTGGCTT GTCCGATTCC GTCATATCTA CC**GTGA**GGCT
GTGANTG10

−741 AATGCCGTTG CGGACTATTT AGCTAGTTAA GGTTTT**TTTT TTCC**CGATTG GAATTCATAC
PYRIMIDINEBOXHVEPB1

−681 TCTTATGAGC CCTCCGAGTG AGGTCATTCC TCTTATTTCA AACGATCACA GGGGCATCGC

−621 CTTGCCCCGT ATGTTGCCTT GTTAGTTTTC CCCCTTTTCA GCAGTTTTTA CGCGAAGGAA

−561 TAAAATTCTT TTTCACCAAA AAAAAAAAAA AAAAAGGATA GTGG**GTGA**AG GTCATTCATA
GTGANTG10

−501 CATCGTTCAC ACATCACCAG ACTTTTTTCG AATTTTATCC GTTTTATTCT GTTGATTCGA

−441 TTACCCATAA GGTAAAGTAA AATTATATTA AACCGGGTTT ATATTGTTAT GATGTTTTTA

−381 TGGGCATTCC CGGCATA**CAC TTG**TCAA**ACG T**C**TACT**TTGT ATA**CTTAAAT ATG**TCAAGTT
MYCCONSENSUSAT ACGTATERD1 CACTFTPPCA1 CArG-box

−321 TCTCATGATT TGTGGTGTTG TATTCAAAGT TAGATATATG TTTTGGTGGC ATTTTGAGAT

−261 GTTT**GTGA**TC AACACTCTGT T**GTGA**GTTCA TTTCAACGA**C AAT**TAATCCG TCTACCGGTA
GTGANTG10 GTGANTG10 CAATBOX1

−201 TTTTTCCGGT TTTT**TGAC**AT ATCAAACGGT GTGC**CACT**TA GTGTGTCTAA CTGATCTCCA
WRKY71OS CACTFTPPCA1

−141 TTATTACAAA AAGGAAAAAG AAGTCAGTTC TCCGT**CACCT ACC**GTT**T**ATT TCAAATTAAC
MYBPLANT

−81 GGCGATCGTA TCCAT**CCAAT** ACTCCAAACG GAAAGTTGAA AGGGTTCTAA TTTAGGGACC
CCAATBOX1 +1

−21 TGGG**GACT**AC TTACTTTCAT C**A**ATTTCTGT ATTGCTTTCA CCAAATTGGC ACCTTTCTCC
CACTFTPPCA1

+40 TTCCCAGTTC T**AGAAA**CCTT TTTCTCTTCC TTTCTTTCTC CCACCTCT**TA CT**CTCTCCAC
POLLEN1LELAT52 CACTFTPPCA1

+100 CTAGTTTTTC TTCCTACTTT GTT**ATG**

Figure S2: ***FaesAP3_2*** **promoter sequence.**

The transcription start site (+1) is in bold and boxed. The start codon ATG is in bold and boxed. Putative cis-acting regulatory elements are in bold and underlined.

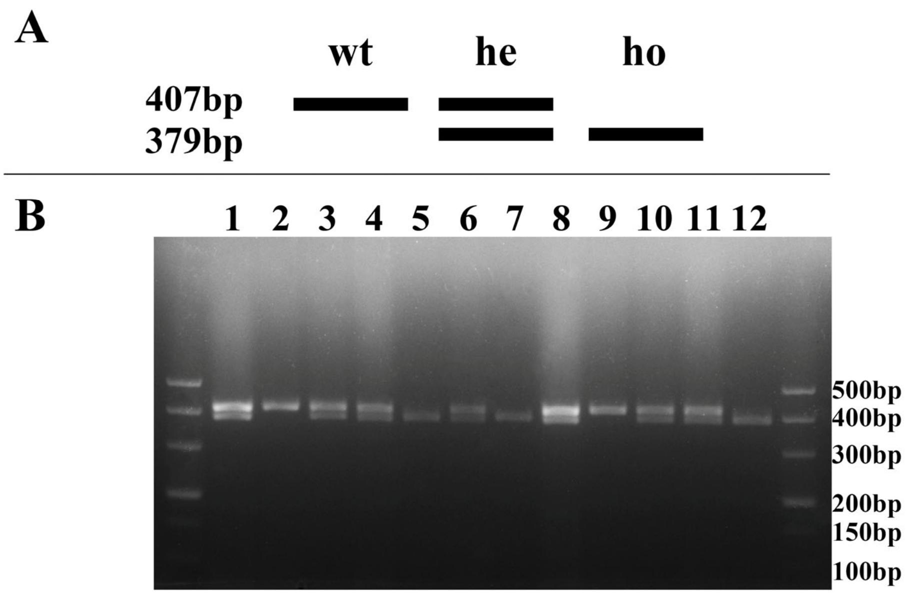

Figure S3. Genotyping of wild-type, heterozygous and homozygous *ap3-3* mutant *A. thaliana* by dCAPS.The amplicons from transgenic lines in wild-type background *Arabidopsis* were cleaved by *Cla*I to produce a 407 bp fragment, and the amplicon from heterozygous *AP3*/*ap3-3* background plants were cleaved by *Cla*I to produce 407 bp and 379 bp fragments; the amplicon from transgenic lines in homozygous *ap3-3* lines were cleaved by *Cla*I to produce a 379 bp fragment.

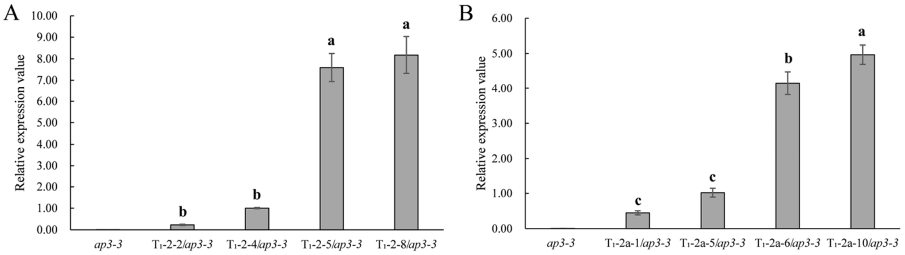

Figure S4. Expression of *FaesAP3_2* and *FaesAP3_2a* in transgenic *Arabidopsis ap3-3* mutant confirmed by qRT-PCR. (A) Expression of *FaesAP3_2* in transgenic *Arabidopsis ap3-3* mutant confirmed by qRT-PCR; (B) Expression of *FaesAP3_2a* in transgenic *Arabidopsis ap3-3* mutant confirmed by qRT-PCR. (*ap3-3*) *Arabidopsis ap3-3* mutant; (T_1-2-2/ap3-3) independent lines of *35S:: FaesAP3_2* transgenic *Arabidopsis ap3-3* mutant with no phenotype complementation; (T_1-2-4/ap3-3) independent lines of *35S:: FaesAP3_2* transgenic *Arabidopsis ap3-3* mutant with a weak complement phenotype; (T_1-2-5/ap3-3) independent lines *35S:: FaesAP3_2* transgenic *Arabidopsis ap3-3* mutant with a medium complement phenotype producing filament attached with carpeloid anther in whorl 3; (T_1-2-8/ap3-3) independent lines *35S:: FaesAP3_2* transgenic *Arabidopsis ap3-3* mutant with strong complement phenotype rescuing stamen-like organs in the 3rd whorl of flower; (T_1-2a-1/ap3-3) independent lines of *35S:: FaesAP3_2a* transgenic Arabidopsis *ap3-3* mutant with no phenotype complementation; (T_1-2a-5/ap3-3) independent lines of *35S:: FaesAP3_2a* transgenic *Arabidopsis ap3-3* mutant with a weak complement phenotype; (T_1-2a-6/ap3-3) independent lines *35S:: FaesAP3_2a* transgenic *Arabidopsis ap3-3* mutant with a medium complement phenotype producing flower with filament attached with stigmatic papillae or carpelloid anther in whorl 3; (T_1-2a-10/ap3-3) independent lines *35S:: FaesAP3_2a* transgenic *Arabidopsis ap3-3* mutant with strong complement phenotype rescuing stamen-like organs in the 3rd whorl of flower. Different letters indicate statistically significant differences.
